# Neuropsychological outcomes in adults commencing highly active anti-retroviral treatment in South Africa: a prospective study

**DOI:** 10.1186/1471-2334-12-39

**Published:** 2012-02-15

**Authors:** John A Joska, Jennifer Westgarth-Taylor, Jacqueline Hoare, Kevin GF Thomas, Robert Paul, Landon Myer, Dan J Stein

**Affiliations:** 1 Department of Psychiatry and Mental Health, University of Cape Town, Cape Town, South Africa; 2 ACSENT laboratory, Department of Psychology, University of Cape Town, Cape Town, South Africa; 3 Department of Psychiatry and Mental Health, University of Cape Town, Cape Town, South Africa; 4 ACSENT laboratory, Department of Psychology, University of Cape Town, Cape Town, South Africa; 5 Department of Psychology and Behavioral Neuroscience, University of Missouri, St. Louis, MO, USA; 6 Centre for Infectious Diseases Epidemiology & Research, School of Public Health & Family Medicine, University of Cape Town and International Centre for AIDS Care & Treatment Programs, Department of Epidemiology, Mailman School of Public Health, Columbia University, Manhattan, USA; 7 Department of Psychiatry and Mental Health, University of Cape Town, Cape Town, South Africa; 8 Department of Psychiatry and Mental Health, Groote Schuur Hospital, Anzio Road, Observatory, Cape Town 7925, South Africa

**Keywords:** HIV neuropsychology, Clade C, Combination anti-retroviral therapy, Neuropsychological outcomes

## Abstract

**Background:**

Infection with HIV may result in significant neuropsychological impairment, especially in late stage disease. To date, there have been no cohort studies of the impact of highly active anti-retroviral treatment (HAART) in South Africa where clade C HIV is predominant.

**Methods:**

Participants in the current study were recruited from a larger study of HIV-associated neurocognitive disorders (HAND) and included a group of individuals commencing HAART (n = 82). Baseline and one-year neuropsychological function was assessed using a detailed battery, and summary global deficit scores (GDS) obtained. Associations with change in GDS were calculated.

**Results:**

Participants had a median CD4 cell count of 166 at baseline and 350 at follow-up. There were significant difference across groups of GDS severity at baseline with respect to level of education and GDS change at one year (*p *= 0.00 and 0.00 respectively). Participants with severe impairment at baseline improved significantly more than those with lesser degrees of impairment. Significant improvements were observed in the domains of attention, verbal fluency, motor function, and executive functions. There were unadjusted associations between GDS change and male gender, lower levels of education, baseline CD4 count and baseline GDS severity. In an adjusted model, only baseline GDS severity (*p *= 0.00) remained significant, with a lower level of education nearing significance (*p *= 0.05). The overall model was highly significant (*p *= 00; r-squared = 0.58).

**Discussion:**

In individuals in late stage HIV commencing HAART in South Africa, those with severe baseline neuropsychological impairment improved significantly more than those less impaired. While improvement across a number of neuropsychological domains was observed, high rates of impairment persisted.

**Conclusions:**

The effects of HAART and participant variables, such as test experience, require clarification. Studies with larger comparison groups, and where HIV disease characteristics are needed to establish whether the trends we identified are clinically meaningful.

## Background

Neuropsychological impairments due to HIV infection of the CNS are detectable across all disease stages, but are more prevalent and marked in individuals with more severe HIV disease [1,2]. Although the use of highly active anti-retroviral therapy (HAART) has halved the incidence of HIV-associated dementia (HAD), the prevalence remains significant [3]. In addition, milder forms of HIV-associated neurocognitive disorders are now known to persist despite the use of HAART [3,4].

Persistent impairment may be the result of several interrelated factors, including host genetic vulnerability, ongoing low grade inflammation within the CNS and the effects of HAART itself [5]. In addition, it has been demonstrated that lower CD4 nadirs are associated with higher rates of neurocognitive impairment [6]. Accordingly, it has been suggested that HAART should be initiated before CD4 counts fall to low levels. The role of HIV subtype or clade is also unclear. The majority of studies investigating HAND and the effects of HAART have been conducted in settings where clade B is predominant, while the burden of HIV disease exists in southern Africa where clade C is prevalent [7]. Pre-clinical studies have suggested that the presence of a defect in the dicysteine motif of the HIV clade C tat protein might reduce the neurotoxic effects of HIV [8]. Clinical reports from both India and South Africa have now established that clinical disorder is in fact highly prevalent, despite this hypothesis [9,10]. There are no studies to date describing the effect of HAART on neuropsychological impairment in South Africa.

Several published studies have reported on neuropsychological improvement following HAART initiation [11-13]. The end-point of treatment is to return neuropsychological function to normal levels following adequate suppression of CNS viral load and related neuro-inflammation [4]. To date, neuropsychological improvement has been associated with effective peripheral viral load suppression, CNS penetration effectiveness (CPE) of HAART regimens, and has been associated with severity of baseline (or study entry) neuropsychological function, and possibly also practice effects in cohort studies [13-16].

The impact of HAART on the CNS has been debated. A significant literature supports the contention that regimens of HAART that are able to penetrate the blood-brain barrier more readily, and in turn adequately suppress CSF viral load, are associated with better neuropsychological outcomes [14,17,18]. However, some literature supports the notion that HAART regimens with higher CNS penetration effectiveness (CPE) may result in neuropsychological impairment [16,19]. Therefore, regarding the role of HAART, it remains to be clarified whether persistent neuropsychological deficits are the result of late-initiation of therapy, the use of lower CPE rank regimens, ongoing low grade neuro-inflammation or HAART-induced neurotoxicity.

The impact of HAART in a prospective study in South Africa where clade C HIV is predominant has not been reported. In this study, we hypothesized that individuals initiating HAART would show improved neuropsychological function over one year. Moreover, we hypothesized that individuals with more severe disease at baseline would have worse neuropsychological outcomes at one year. Domain-specific changes were also explored.

## Methods

### Subjects

This study was conducted as part of a larger investigation of HIV-associated neurocognitive disorders (HAND) in Cape Town, South Africa that has been described previously [20]. In summary, we conducted detailed neuropsychological assessments on 166 participants recruited from three primary health care centres. At each visit, detailed socio-demographic and neuromedical measures were also administered and laboratory tests completed. Participants were included if they were soon to commence treatment with HAART according to South African guidelines. They were excluded if they had a severe psychiatric disorder (such as schizophrenia or bipolar disorder), recent (within the last three months) history of substance abuse, or significant neurological disorder (such as epilepsy or significant head injury defined as a loss of consciousness for more than 30 mins). We were able to re-assess 104 participants at a one year follow-up assessment. Of this group, 22 did not initiate HAART for the following reasons- one participant had one month of treatment before being imprisoned and defaulting. Of the remaining 21, 15 had CD4 cell counts above the guideline for initiation and were not enrolled onto HAART. Their CD4 cell counts were unknown at the time of initial study recruitment. The other six participants qualified for HAART, and were included in the study, but had not attended clinic appointments during the one-year period and so were not initiated on treatment.

Normative data for neuropsychological testing of isiXhosa-speaking participants was obtained from 120 HIV- negative participants recruited from Voluntary Counseling and Testing services located at the same primary care clinics. Other than being HIV negative, as confirmed by a recent rapid HIV test and confirmatory serological test, inclusion and exclusion criteria were identical to the HIV-positive participants.

The CD4 cell counts were extracted from the laboratory records, linked by the participants' clinic numbers. Clade sequencing was not available on this sample but more than 90% of infected individuals in the Cape Town area are infected with clade C virus [21]. Hepatitis sero-status was not established but the prevalence of hepatitis C in South Africa is extremely low [22] The use of HAART was also extracted post-hoc from clinical records, and the CPE rank for each was calculated using previously published criteria [14].

All participants who met study criteria and agreed to participate provided written informed consent. Approval to conduct the study was obtained from the Research Ethics Committee of the Faculty of Health Sciences, University of Cape Town, and from the relevant health authorities.

### Neuropsychological test battery

A neuropsychological test battery was administered to all participants to assess specific domains of neurocognitive function. The battery comprised tests of the domains: attention (the Mental Alternation Test (MAT) and the Mental Control Test (MCT)), learning and memory (the Hopkins Verbal Learning Test (HVLT) and the Brief Visuospatial Memory Test (BVMT)), psychomotor speed (Finger tapping (FT) and the Grooved Pegboard Test (GP)), psychomotor speed (Trail Making Test part A (TMTA), Color Trails Test 1(CT1) and Digit Symbol-Coding (DSC)), executive function (Color Trails Test 2 (CT2), Stroop Colour-Word test (SCW), Wisconsin Card-Sorting Test (WCST), and language (category fluency)).

### Determination of global neuropsychological function

Raw data from the above neuropsychological test battery were converted to t-scores for each test using the demographically-adjusted normative data. From these, a deficit score for each test was obtained using the methods previously reported by Heaton and Carey [1,23]. The deficit score ranges from 0 through 5. A score of 0 is obtained for t-scores in the normal or superior range, minimizing the effect of averaging out of impairments in other domains. This provides a more sensitive method for generating a summary neuropsychological score than averaging neuropsychological scores. An average global deficit score calculated (GDS) is then obtained for each participant, with higher scores indicative of greater impairment. GDS scores for participants at baseline and follow-up were generated, and the change in GDS calculated. GDS scores provide a continuous measure of impairment with scores of > 0.5 providing high rates of specificity and positive predictive value in establishing HIV-associated impairment. In order to establish whether the degree of severity at baseline was associated with demographic, clinical or disease variables, we group the GDS into a non-impaired group (scores < 0.25), a mild-moderately impaired group (scores > 0.25 to < 0.75) and a severely impaired group (scores > 0.75).

### Statistical analysis

Data were analysed using STATA 11 (Stata Corporation, Texas, USA). As data were not normally distributed across all variables, we calculated medians and interquartile ratios for the various cohort groups, and for the GDS severity group. Comparisons were made using Wilcoxon rank sum tests or Fisher Exact tests where appropriate. We generated line plots for the two time-points of the three GDS HAART-treated groups, and compared mean summary scores using Kruskal Wallis tests. Impairment in individual domains (calculated as a GDS score of ≥ 0.5) were used to generate rates of impairment at baseline and follow-up. Differences in proportions across the time-points were calculated using McNemar's chi-square test. We then evaluated linear trends first using unadjusted models across the participants, with change in GDS between follow-up and baseline as the dependent variable. We included independent variables in a final multiple linear regression, if the simple model rendered a p-value of less than 0.1, or the variable was known to be strongly associated with neuropsychological test performance. The final model included gender, education, baseline and follow-up CD4 cell count, and baseline GDS.

## Results

A total of 166 participants were recruited into the cohort study. Of these, 58 were not re-assessed at one year for reasons including death (n = 9), migration (n = 6) and clinic loss-to-follow-up (n = 33). Of the 108 participants retained and who completed neuropsychological (NP) batteries at the two time points, 26 did not receive HAART and were not included in the cohort analysis. The three groups (non-retained, retained with no HAART and retained with HAART) were compared with respect to demographic and disease characteristics and there were no significant differences across groups (Table 1). Of note, there was no difference in their baseline GDS score (*p *= 0.14).

**Table 1:** Demographic, disease and neuropsychological characteristics of all participants from baseline

	Non-retained (n = 58)	Retained no HAART (n = 26)	Retained with HAART (n = 82)	*p*-value
**CD4 baseline median (IQR)**	165 (84-218)	192 (172-329)	166 (119-211.5)	0.1
**Women (%)**	40 (70)	19 (73)	64 (78)	0.58
**isiXhosa**	46 (81)	24 (83)	75 (91)	0.48
**Age median (IQR)**	30 (27-32)	30 (27-33)	29.5 (27-32)	0.39
**Education median (IQR)**	10 (9-11)	10 (9-11)	10-9-12)	0.69
**Baseline total GDS median (IQR)**	0.67 (0.27-1.33)	0.5 (0.27-1.0)	0.47 (0.13-0.93)	0.14

**Figure 1:**
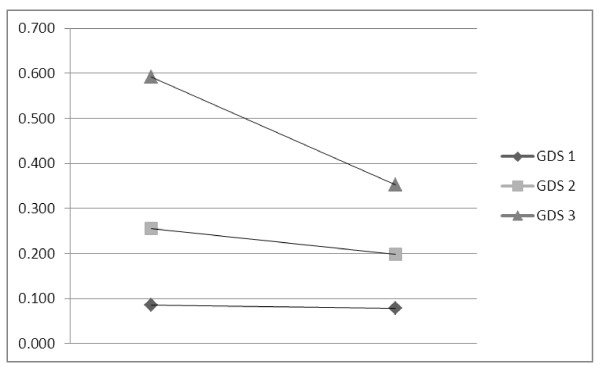
**Change in Global Deficit Score at baseline and one year of participants initiating HAART across groups of severity at baseline (n = 82). Mean change: GDS 1 = 0.01; GDS 2 = 0.06; GDS 3 = 0.24. Kruskal Wallis *p*-value = 0.00**. Percent neuropsychological impairment over one year by domain. Black bars: Baseline data; Hatched bars: One year data.

Demographic, disease and neuropsychological characteristics of participants initiating HAART across groups of baseline GDS severity are presented in Table 2. The mean change in GDS between assessments for all participants was 0.13, and the improvement was highly significant (*p *= .00). There were no differences across groups for gender, home language, age, baseline CD4 count, duration of HAART or CPE. The groups differed for level of education, with the groups of increasing severity having a lower level of education (*p *= .00). The groups also differed with respect to change in GDS, with those more severely affected at baseline showing a greater degree of change in GDS (*p *= .00). The extent of GDS change is presented in Figure 1.

**Table 2:** Characteristics of participants with HAART over one year grouped by total GDS severity

		GDS Groups		
	1	2	3	*p*-value
Number Women (%)	21 (80.7)	21 (84)	21 (67.7)	0.34
Number isiXhosa (%)	22 (88.5)	23 (95.7)	30 (96.8)	0.22
Age median (IQR)	30 (27-31)	28 (25-32)	30 (28-32)	0.34
Education median (IQR)	12 (10-12)	10 (10-12)	9 (8-10)	0.00*
CD4 One median (IQR)	180 (146-199)	182.5 (143-221)	138 (100-205)	0.32
CD4_Two median (IQR)	395 (271-465)	340 (263-518)	302 (156-388)	0.07
Duration of HAART in months median (IQR)	11 (10-12)	11 (10-14)	10 (8-12)	0.45
CPE median (IQR)	2 (1.5-2.5)	2 (1.5-2.25)	2 (1.5-2.5)	0.98
GDS Change median (IQR)	0 (-0.67-0.67)	0.13 (-0.67-0.27)	0.6 (0.2-0.8)	0.00*

**Figure 2:**
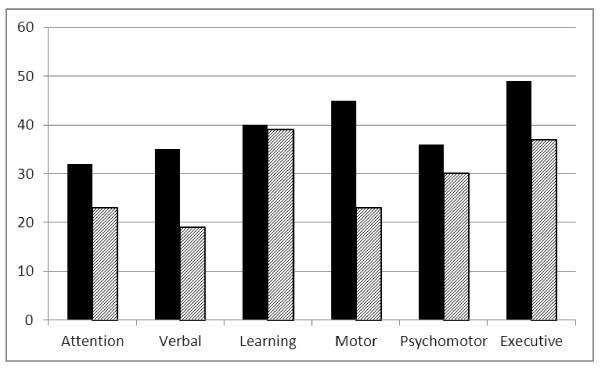
**Percent neuropsychological impairment over one year by domain**. Black bars: Baseline data; Hatched bars: One year data

The rates of impairment across neuropsychological domains at baseline and follow-up are shown in Figure 2. There were significant improvements in the proportion of impairment for the domains of attention (*p *= .01), verbal fluency (*p *= .00), motor speed (*p *= .00) and executive functioning (*p *= .02). No improvement was noted for learning (*p *= .83) and psychomotor processing (*p *= .12). High rates of impairment persisted at one year (range 23-45%).

Potential predictors of GDS change were examined using a series of linear regression models. There were significant unadjusted associations between male gender (*p *= .01), having a lower level of education (*p *= .00), baseline CD4 count (*p *= .04) and baseline GDS severity (*p *= .00) with GDS change (Table 3). When these associations were entered in a final adjusted model, only baseline GDS severity remained significantly associated with GDS change (*p *= .00), while a lower level of education neared significance in the model (*p *= .05). The overall model was highly significant and provided a moderate level of predictive value (*p *= .00; r-squared = 0.58). There was no effect of the duration of HAART or CPE on GDS change. In order to investigate potential associations between demographic and disease characteristics on baseline GDS, we examined unadjusted associations between these demographic and clinical variables and baseline GDs severity. Only a lower level of education was significantly associated with GDS (*p *= .00; coefficient:-0.16; 96%CI:-0.22-0.10).

**Table 3:** Unadjusted and adjusted regression models for predictors of change in GDS at one year on HAART

	Unadjusted associations			Adjusted associations		
	Regression coefficient	95% CI	*p*-value	Regression coefficient	95% CI	*p*-value
**Age**	0	-0.05	0.96	-	-	-
**Male gender**	0.24	0.05-0.43	0.01*	0.09	-0.34	0.09
**Education**	-0.07	-0.09	0.00*	0.05	-0.11	0.05
**Baseline CD4**	0	0	0.04*	0	-0.00-0.00	0.46
**Baseline GDS**	0.51	0.40-0.62	0.00*	0.57	0.40-0.62	0.00*
**Duration of HAART**	0.01	-0.03	0.38	-	-	-
**Follow up CD4**	0	-0.03	0.05	0	0	0.71
**CPE**	0.03	-0.35	0.69	-	-	-

Overall *p*-value 0.00*; R-squared 0.58

## Discussion

We report on the first descriptive follow-up study conducted among a group of patients utilizing a detailed neuropsychological battery to establish the effects of HAART in South Africa where clade C HIV is predominant. We found significant improvement in individuals commencing HAART at one year follow-up. There was improvement in most, but not all neuropsychological domains, with high rates of impairment persisting. Improvement in neuropsychological function was most marked for those with a lower level of education and a greater degree of impairment at baseline. Unadjusted associations with GDS change were noted for male gender, a lower level of education, lower baseline CD4 count and a greater baseline GDS severity. Individuals most impaired at baseline were more likely to improve at one year than those less impaired.

The use of HAART has frequently been reported as improving neuropsychological outcomes in prospective cohorts [3,13,24,25]. Predictors of improvement previously reported include higher CD4 count nadir, high CPE, stability of HAART regimen, lower levels of baseline impairment and good medication adherence [26,27]. All participants in this study had CD4 counts less than 200 cells/ml due to existing South African HAART guidelines; this might account for high rates of impairment at baseline, and to some extent, to persistence at follow-up. It is possible that we did not detect an effect for low CD4 count as the value range was limited to 119 to 215 in this cohort. We did not gather data on nadir CD4 count specifically, although in most instances, the value obtained at this pre-HAART visit is the nadir. We did not detect an effect of different CPE ranks, as first-line regimens, used by the majority of participants in our study have moderate to high CPE ranks (73 of 82 participants had a CPE rank ≥ 1.5). Also, almost all participants achieved peripheral viral load suppression during the first year of treatment, and therefore were adequately immune reconstituted. This suggests that they were also adherent to treatment.

There are few data enumerating changes in neuropsychological performance by domain. Some have shown improvements in psychomotor speed [12,28], while others have reported either improvement in global scores or across multiple domains [29-31]. While we report on improvement across most domains of function, we did not observe this for learning or psychomotor processing. The domain of learning and psychomotor processing has been held to be a core feature of HIV-associated neurocognitive impairment, due to its predilection for the deep grey nuclei [32]. The absence of significant improvement suggests that HIV-associated disease is either established earlier and therefore less amenable to reversal by HAART in late stage/low CD4 nadir disease; or that neuro-inflammation is ongoing in these brain regions; or lastly, that HAART is not effective in these areas due to deficient penetration or a direct drug toxicity.

In the neuropsychology literature, improvement is usually associated with less baseline impairment [33]. We noted the converse, that more severe baseline performance was associated with most improvement. A greater degree of GDS change was significantly associated with male gender, a lower level of education, baseline CD4 cell count, baseline GDS severity and neared significance with follow-up CD4 cell count (*p *= 0.05). Only baseline GDS severity remained significant in adjusted models, while a lower level neared significance (*p *= 0.05). The strong trend to improvement in those most severely affected likely is explained by the range of possible improvement being wider; those with less impairment probably experienced a ceiling effect on their ability to improve. The improvement shown by individuals with less education in the adjusted model suggests that this effect was present together with the degree of impairment. This effect may be explained by participant characteristics such as test experience, whereby individuals with lower education benefitted more from the experience of being tested. In South Africa, the effects of quality of education, as opposed to levels of education, have been shown to exert effects in test settings [34].

It is less well understood whether improvement in cohort studies is associated only with a HAART effect, or is also associated with practice effect, test familiarity or other participant characteristics, such as level and quality of education [32,4]. We re-assessed participants after one year to reduce practice effect, although some degree of familiarity with test environment and administration may have had an impact on improvement. When we investigated the associations of baseline GDS, we noted only lower education to be a significant predictor. In this cohort, the medians across the GDS severity groups ranged from 9-12 years which might have had a substantial effect on performance.

## Conclusions

Several limitations of our study warrant discussion. We were able to retain a smaller sample of participants than originally recruited. The significant drop-out rate is due to a combination of factors, including large patient numbers at primary care clinics making tracking and retention by services difficult, mortality during early treatment, and migration of individuals. We also did not include additional groups of differently defined HIV positive participants who may have acted as controls. Such groups may have allowed for comparison of possible practice effects. These control groups may have included participants on stable HAART regimens, or those who were not. In the latter case, it would not be ethical to withhold treatment from individuals who met criteria for HAART. This group would therefore by definition have a different set of disease characteristics. We did not perform HIV subtype analysis on this sample, which would have allowed us to draw clearer conclusions about the putative role of clade in neuropathogenesis. Also, we were not able to obtain CSF in order to characterize intra-thecal viral load or inflammatory markers. The use of lumbar for research has not been approved widely in our setting. Lastly, we did not control for the range of baseline neuropsychological impairments we detected. The inclusion of a non-impaired group in measurement of change does affect group differences and outcomes.

We believe that this study makes an important contribution to our understanding of the effect of HAART on neuropsychologically impaired individuals, in South Africa, where clade C HIV is highly prevalent. This benefit seemed to accrue most to individuals with more severe baseline impairment. Studies with larger comparison groups, and where HIV disease characteristics are similar, are needed to establish whether the trends we identified are clinically meaningful.

## Competing interests

The authors declare that they have no competing interests.

## Authors' contributions

JJ conceived and led the study, conducted data analysis and write the paper. JWT supervised neuropsychological assessments and scoring. JH assessed participants for neurologic disease. KT, RP and DJS participated in the design of the study and overall management. LM provided statistical consultancy. All authors read and approved the final manuscript.

## Pre-publication history

The pre-publication history for this paper can be accessed here:

http://www.biomedcentral.com/1471-2334/12/39/prepub
